# Genome-wide identification and expression analysis of the NCED family in cotton (*Gossypium hirsutum* L.)

**DOI:** 10.1371/journal.pone.0246021

**Published:** 2021-02-25

**Authors:** QingHua Li, XianTao Yu, Long Chen, Gang Zhao, ShiZhou Li, Hao Zhou, Yu Dai, Na Sun, YongFei Xie, JunShan Gao, DaHui Li, Xu Sun, Ning Guo

**Affiliations:** College of Life Sciences, Anhui Agricultural University, Hefei, Anhui, China; University of Hyderabad, INDIA

## Abstract

Abscisic acid (ABA) is an important plant hormone that plays multiple roles in regulating growth and development as well as in stress responses in plants. The *NCED* gene family includes key genes involved in the process of ABA synthesis. This gene family has been found in many species; however, the function of the *NCED* gene family in cotton is unclear. Here, a total of 23 *NCED* genes (designated as *GhNCED1* to *GhNCED23*) were identified in cotton. Phylogenetic analysis indicated that the identified NCED proteins from cotton and *Arabidopsis* could be classified into 4 subgroups. Conserved motif analysis revealed that the gene structure and motif distribution of proteins within each subgroup were highly conserved. qRT-PCR and ABA content analyses indicated that *NCED* genes exhibited stage-specific expression patterns at tissue development stages. *GhNCED5*, *GhNCED6* and *GhNCED13* expression was similar to the change in ABA content, suggesting that this gene family plays a role in ABA synthesis. These results provide a better understanding of the potential functions of *GhNCED* genes.

## Introduction

Abscisic acid (ABA) plays an important role in the process of plant growth, development and stress tolerance [1]. In plants, ABA can be synthesized directly or indirectly, and the indirect C40 pathway for ABA synthesis is utilized in most higher plants [2]. In the C40 pathway, zeaxanthin is converted to violaxanthin by zeaxanthin epoxidase (ZEP) and then converted to neoxanthin. Violaxanthin and neoxanthin are cleaved into xanthoxin by catalysis of 9-cis-epoxycarotenoiddioxygenase (NCED) [3], and xanthoxin is converted to abscisic aldehyde by short-chain alcohol dehydrogenase (ABA2). Finally, abscisic aldehyde is oxidized to ABA by aba-aldehyde oxidase (AAO).

9-Cis-epoxycarotenoid dioxygenase (NCED) has been identified in many plants and is a rate-limiting enzyme for ABA biosynthesis. Early research indicated that a slight divergence occurred in the NCED subfamily and the exon position is highly conserved across diverse lineages. A slow evolutionary rate occurred in *NCED* genes, which might facilitate tissue-specific functional divergence in NCED subfamilies [4].

The NCED gene was first cloned in the maize ABA-deletion mutant Vp14 [3, 5]. Furthermore, nine *NCED* genes have been identified in *Arabidopsis*, and five of these genes (*AtNCED*2, *AtNCED3*, *AtNCED5*, *AtNCED6*, and *AtNCED9*) are related to ABA synthesis [6]. The study demonstrated that improved drought tolerance by manipulation of the *AtNCED3* gene results in the accumulation of endogenous ABA [7]. Subsequently, *NCED* genes have been identified in *Tamatim* [8], cowpea [9] and rice [10].

Cotton (*Gossypium hirsutum l*.) is one of the most widely cultivated and important crops in the world [11]. Upon completion of whole-genome sequencing of cotton, the whole-genome sequence of the upland cotton genetic standard line TM-1 was recently published [12, 13]. However, investigations of *NCED* genes from cotton are limited. The role of the NCED family in cotton development has not been clearly identified. We studied the whole genome of upland cotton and identified all the potential *GhNCED* family genes using bioinformatics analysis. We identified three genes related to the change in ABA content during cotton seedling growth and development. The findings of this study provide new insights into functional differentiation and evolution of the *NCED* gene family in plants.

## Materials and methods

### Plant materials and genomic database

Upland cotton TM-1 was provided by the Beijing Academy of Agricultural Sciences. Cotton materials were cultured in light incubator at 21˚C with 16 h of light and 8 h of darkness per d. The samples were collected at 15, 18, 21, 24, and 27 d and stored at -80˚C for further analysis. The genome information of cotton were derived from the database (http://mascotton.njau.edu.cn), and the genome data of Arabidopsis were derived from the database (http://www.arabidopsis.org/).

### Identification of the NCED gene family in cotton

BioEdit software was used to establish a local database using the genome-wide amino acid (AA) sequence of upland cotton as a data source. The AA sequences of *A*. *thaliana* NCED proteins were used as query sequences, and the BLASTP algorithm (E-value = 0.001) in the established local database of upland cotton was used to search for *NCED* genes in cotton. To confirm members of the upland cotton NCED family, a hidden Markov model of the NCED domain was used in the Pfam (http://pfam.wustl.edu/hmmsearch.shtml) (E-value = 0.001) and SMART (http://smart.embl-heidelberg.de) databases. The ClustalW tool in MEGA5.0 software [14] was used to conduct multiple sequence alignment of the obtained *NCED* gene sequences to remove repeat sequences. The obtained AA sequences were analyzed online using ExPAsy (http://www.expasy.org/) to determine the physical parameters of the *GhNCED* proteins, including their isoelectric points (PIs) and molecular weights (MWs).

### Conserved motifs and gene structure analysis of the *GhNCED* gene family

Multiple Expectation Maximization for Motif Elicitation (MEME) (http://meme.sdsc.edu/meme4_3_0/intro.html) was used to analyze the conserved motifs of NCED gene family proteins in upland cotton [15]. The maximum value of the motif was set to 30, and the length of the motif was set between 6 and 200. Then, the TBtools tool was used to visualize the conserved motifs. Gene Structure Display Server (GSDS, http://gsds.cbi.pku.edu.cn/) analytical tools were used for visualization of the exon substructure of NCED family genes.

### Phylogenetic analysis of the *NCED* gene family in cotton and *Arabidopsis*

The ClustalW algorithm provided in MEGA 6.0 software was used to conduct multisequence alignment of the AA sequence of the *NCED* genes in upland cotton and *Arabidopsis*. All the NCED protein sequences were downloaded from the NCBI database (https://www.ncbi.nlm.nih.gov/). The phylogenetic tree was constructed by using the neighbor-joining (NJ) method with 1000 bootstrap test.

### Chromosome localization of the *GhNCED* gene family

The starting position of each NCED gene on the chromosome was obtained from the upland cotton genome database. The chromosomal locations of these genes were mapped using MapInspect software (http://mapinspect.software.informer.com).

### ABA extraction and HPLC analysis

Cotton seedlings were grown in a light incubator in the laboratory. The first true leaves from seedlings germinated at 15 d (0 d). ABA was extracted from cotton leaves every 3 d (15, 18, 21, 24 and 27 d) and detected by high-performance liquid chromatography (HPLC). For ABA extraction, approximately 1 g of cotton leaves was weighed and extracted with methanol at 4°C for 12 h. Each mix was centrifuged for 15 min at 5000 rpm, and the supernatants were then stored at 4°C. Each precipitate was also extracted with methanol twice. Polyvinylpolypyrrolidone (PVPP) was added to adsorb phenolics and pigments from the resulting supernatants. Each sample was oscillated (200 rpm for 1 h, at 4°C) and frozen in a cryogenic refrigerator (-80°C) to form a frozen body. The frozen bodies were thawed (4°C) and then centrifuged (10000 rpm for 15 min). Each precipitate was washed with methanol three times and then rotary-evaporated to dryness. The resulting precipitates were resuspended in methanol and were filtered to produce filtrates for HPLC detection (UV 210 nm, 1 mL/min).

### Expression analysis of the *GhNCED* gene

The qRT-PCR primers are listed in S1 Table. Total RNA was extracted from upland cotton leaves at different developmental stages using TRIzol Reagent. cDNA synthesis was performed using an RT reagent kit (Tiangen, China). qRT- PCR was analyzed in a 20-μL reaction system (including 10 μL SYBR ® Premix Ex Taq ™ Ⅱ (2 x), 2 μL cDNA, and 0.8 μL upstream and downstream primers) and a simple procedure (50°C for 2 min; 40 cycles at 95°C for 30 s, 95°C for 5 s, and 60°C for 20 s; and a final extension at 72°C for 10 min). The *GhUBQ* gene was used as a control, and each sample was repeated 3 times. The relative expression levels were calculated using the 2^−Ct^ method. All experiments included three biological replicates.

## Results

### Identification of the *GhNCED* gene family

To identify the NCED gene family of *G*. *hirsutum*, we performed BlastP searches against the cotton genomics databases using the NCED domain in *Arabidopsis thaliana*. A total of twenty-three NCED family members were identified from *G*. *hirsutum*, and these genes were provisionally named *GhNCED1* to *GhNCED23* (Table 1). In addition, the basic information for all the *GhNCED* genes was obtained from the ExPAsy online website, and the lengths, MWs, PIs and chromosomal locations of the *GhNCED* genes are listed in Table 1. The results showed that MW exceeded 50 for all the *GhNCED* genes except for *GhNCED14* and *GhNCED19*. In addition, most of the *GhNCED* genes had PI values less than 7, indicating that the proteins encoded by the *GhNCED* genes are weak acids and that these proteins may play a role in cells in an acidic environment.

**Table 1:** Information for the *GhNCED* gene family in cotton.

Gene	Protein ID	Chromosome Localization	ORF	Number of AAs	MW	PI
GhNCED1	CotAD_25649	9	1821	607	67.64	6.06
GhNCED2	CotAD_75526	scaffold	1818	606	67.71	6.41
GhNCED3	CotAD_45615	scaffold	1806	602	67.29	6.27
GhNCED4	CotAD_27475	7	1518	506	56.61	5.59
GhNCED5	CotAD_41298	10	1806	602	67.26	7.35
GhNCED6	CotAD_04169	5	1716	572	63.26	6.06
GhNCED7	CotAD_45595	scaffold	1791	597	65.47	6.53
GhNCED8	CotAD_14690	scaffold	1716	572	63.12	6.00
GhNCED9	CotAD_13950	13	1767	589	65.19	5.96
GhNCED10	CotAD_09370	7	1767	589	65.19	6.10
GhNCED11	CotAD_35755	scaffold	1791	597	65.64	6.60
GhNCED12	CotAD_11450	6	1806	602	67.45	8.78
GhNCED13	CotAD_67889	scaffold	1806	602	67.52	8.94
GhNCED14	CotAD_26725	11	1059	353	38.99	8.81
GhNCED15	CotAD_42837	8	1815	605	66.41	6.71
GhNCED16	CotAD_17632	8	1806	602	66.32	6.95
GhNCED17	CotAD_44591	6	1509	503	57.12	8.32
GhNCED18	CotAD_34311	11	1899	633	71.03	6.24
GhNCED19	CotAD_27691	9	1221	407	46.09	7.01
GhNCED20	CotAD_00217	9	1812	604	67.55	6.01
GhNCED21	CotAD_34301	11	1680	560	62.87	6.53
GhNCED22	CotAD_00216	9	1710	570	64.23	5.97
GhNCED23	CotAD_22927	10	1701	567	63.18	6.25

### Genetic structure and phylogenetic evolution of the *GhNCED* family

**Fig 1:**
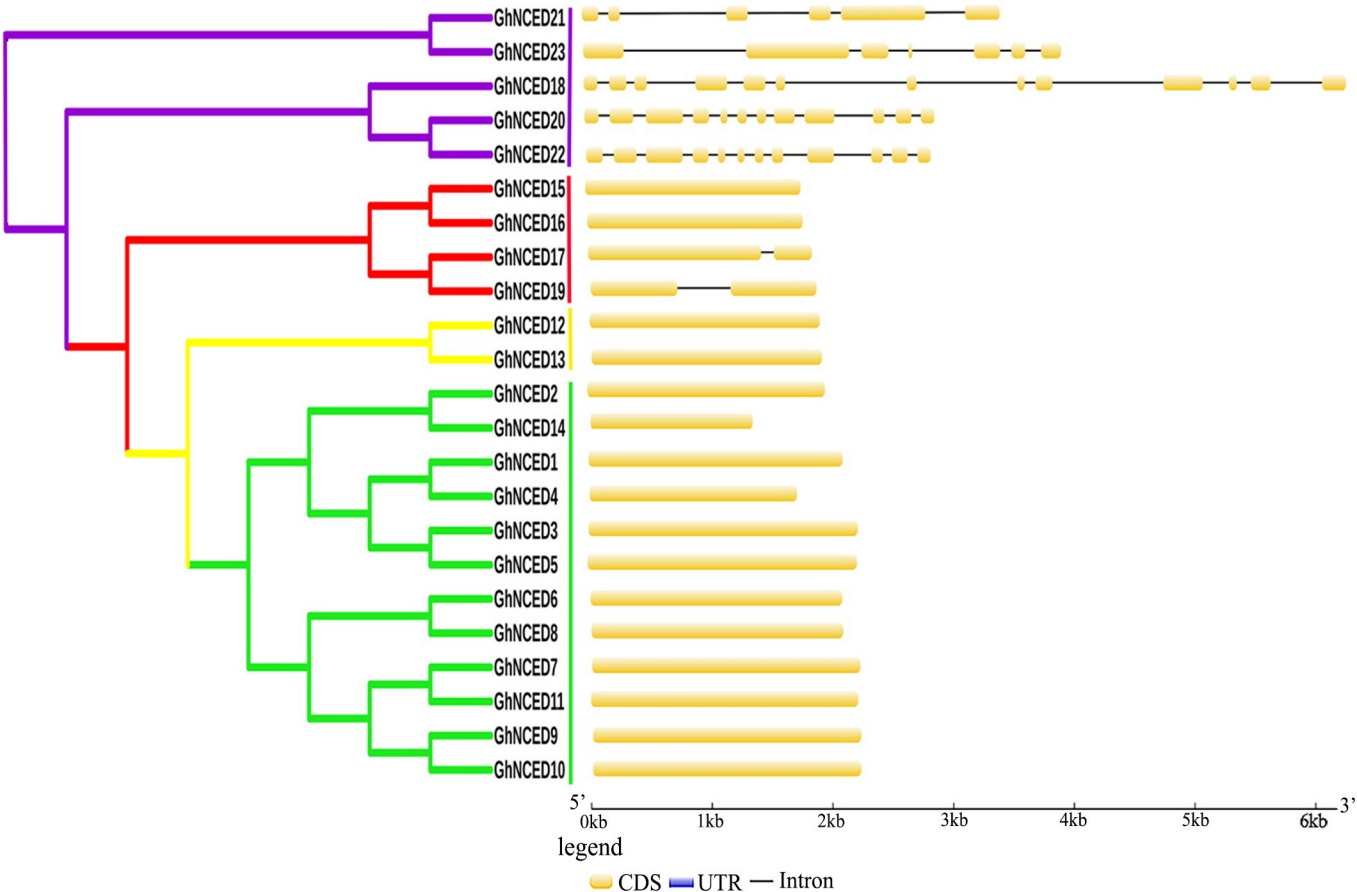
**Cotton NCED family phylogenetic tree (left) and gene structure (right).** Phylogenetic relationships among GhNCED proteins were generated using the NJ method with the JTT model in MEGA 5.1. The exon-intron organization of the GhNCED gene was plotted using the GSDS database. The 23 GhNCED proteins were classified into four classes: I, II, III, and IV (left). The blue boxes in the two terminals represent the 5’-UTR and 3’-UTR, and the yellow boxes indicate exons. The separate black lines between exons represent introns.

According to the structural features of GhNCED proteins, the *GhNCED* genes can be divided into four subgroups (Fig 1). We analyzed the motif distribution using the MEME web server. Five genes (*GhNCED21*, *GhNCED23*, *GhNCED18*, *GhNCED20* and *GhNCED22*) contained 5–12 introns, whereas two genes (*GhNCED17* and *GhNCED19*) contained only one intron each. The remaining GhNCED genes had no introns; each only had a single coding sequence. Clustering analysis revealed that these genes were in the same branch, indicating that their functions are highly consistent.

### Analysis of the phylogenetic relationships of the *GhNCED* genes

**Fig 2:**
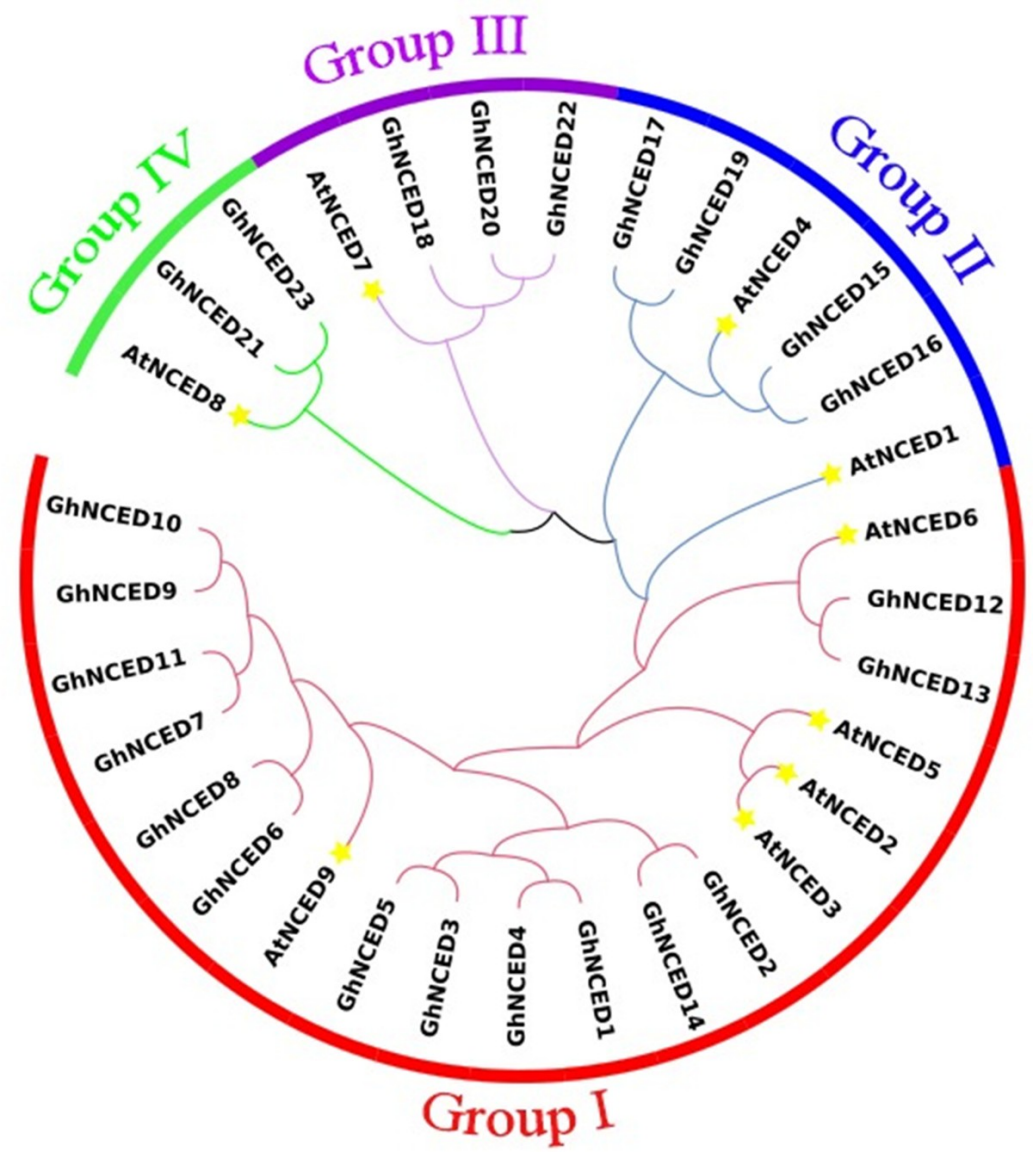
Clustering analysis of NCED proteins. Phylogenetic tree of NCED proteins in *Gossypium hirsutum L* and *Arabidopsis*. The unrooted tree was also constructed using the NJ method with the JTT model in MEGA software. The parameters were 1000 bootstraps and pairwise gap deletion. The 32 NCED proteins were classified into four groups: I, II, III, and IV.

**Fig 3:**
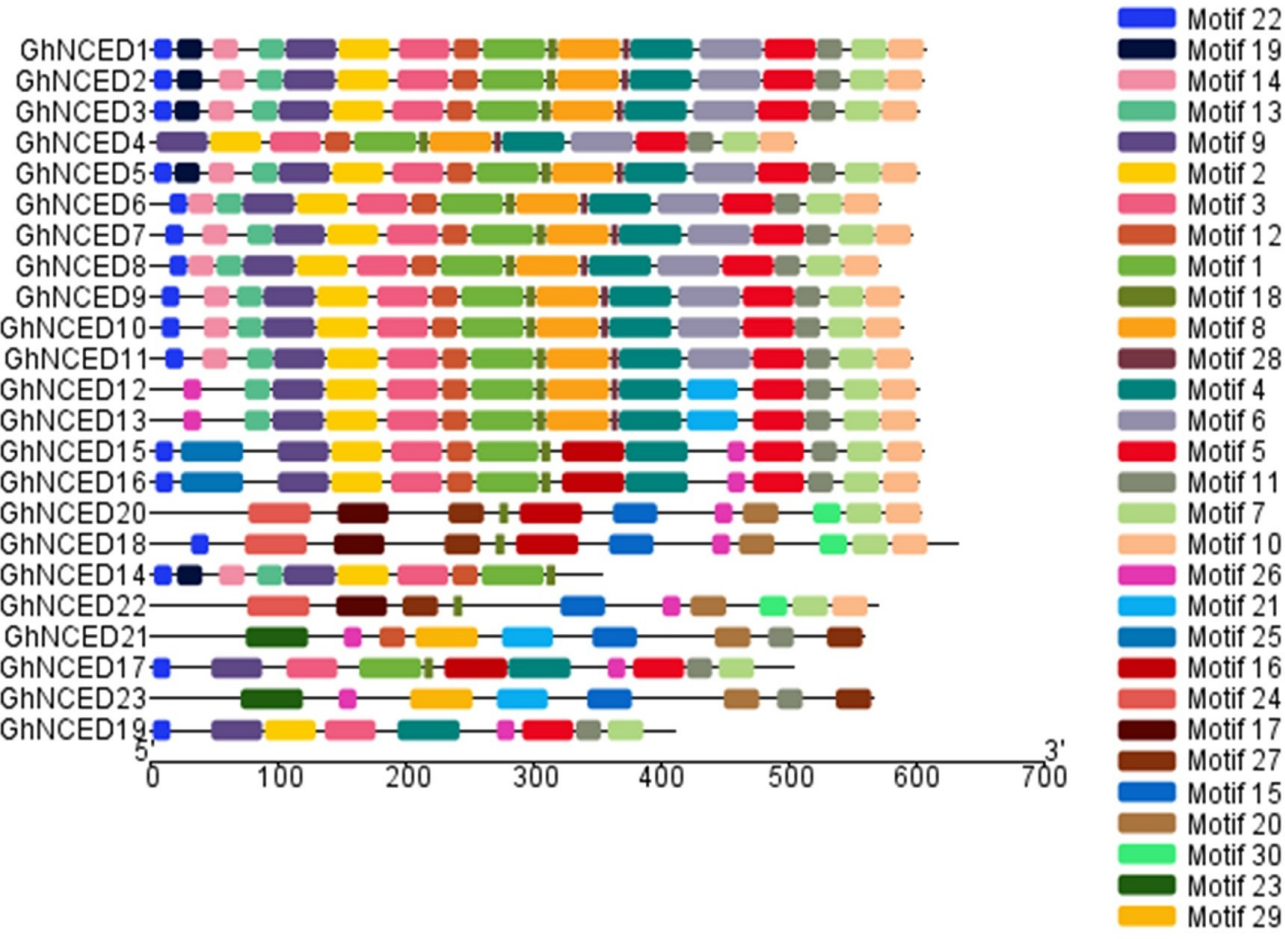
Conserved domains in GhNCED proteins. Domains were determined through searching the GhNCED protein sequences by MEME, and 30 conserved domains were found.

The phylogenetic tree of NCED proteins from *G*. *hirsutum* and *Arabidopsis thaliana* was constructed with Mega6.0 software (Fig 2). There were 23 candidate Gh*NCED* proteins from *G*. *hirsutum* and 9 AtNCED proteins from *Arabidopsis thaliana*. Through comparison of the sequence features of their NCED proteins (Fig 3), we found that the NCED genes in upland cotton and *Arabidopsis* could be divided into four subgroups: Group Ⅰ, Group Ⅱ, Group Ⅲ and Group Ⅳ. We found 5 *AtNCED* genes (*AtNCED2*, *AtNCED3*, *AtNCED5*, *AtNCED6* and *AtNCED9*) and 14 GhNCED genes (*GhNCED1* to *GhNCED14*) that were directly related to ABA synthesis, and these genes clustered together in Group Ⅰ. *AtNCED1* and *AtNCED4* as well as *GhNCED15*, *GhNCED16*, *GhNCED17* and *GhNCED19* were clustered together in Group Ⅱ. *AtNCED7*, *GhNCED18*, *GhNCED20* and *GhNCED22* were clustered together in Group Ⅲ. The remaining three genes (*AtNCED8* with *GhNCED21* and *GhNCED23*) were clustered together in Group Ⅳ.

### Analysis of the conserved motifs of the *GhNCED* gene family

**Fig 4:**
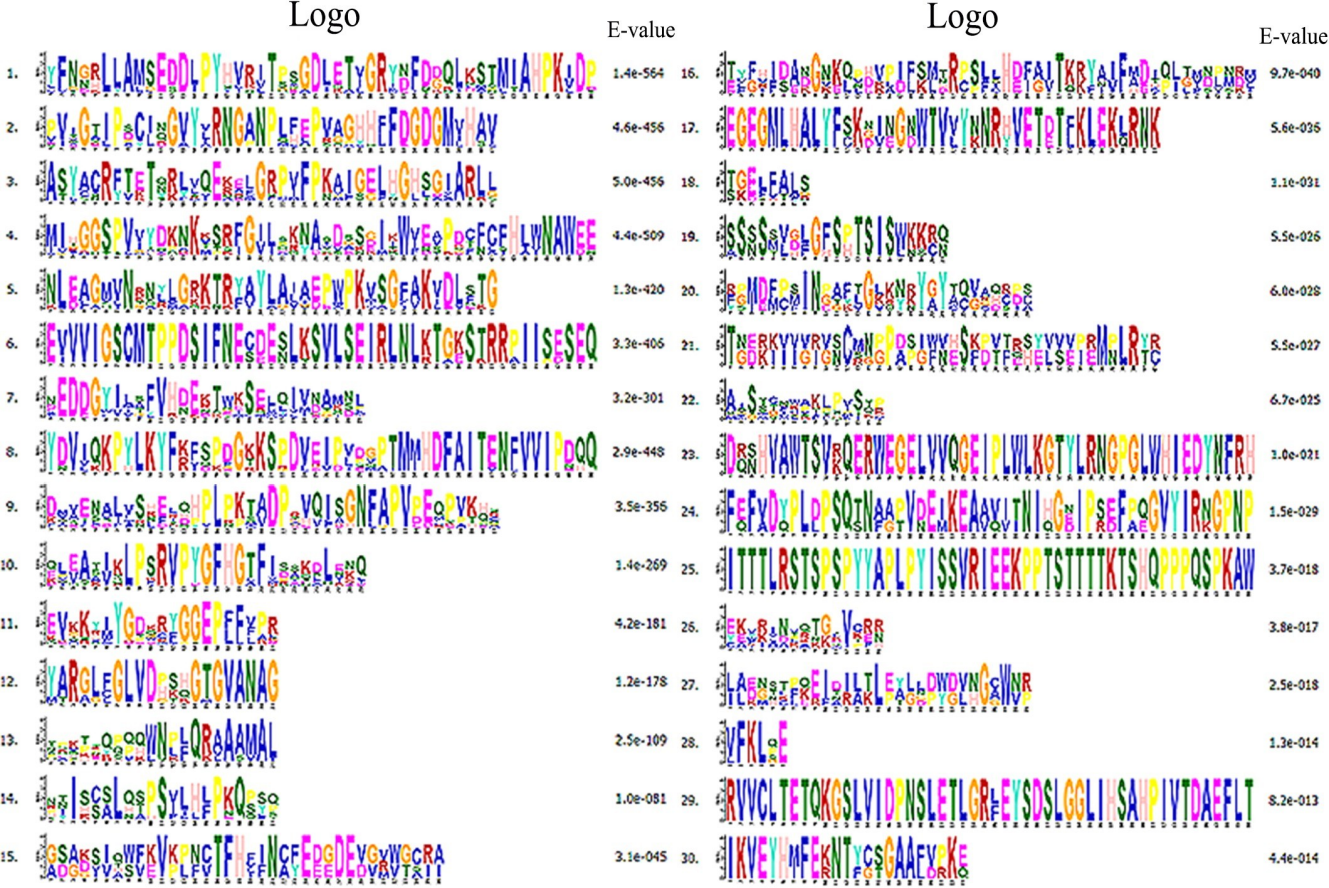
Distribution of the conserved motifs of NCED proteins in *G*. *hirsutum* L.

Thirty conserved motifs of NCED proteins in upland cotton were identified using the MEME web server (Fig 4). The types and numbers of motifs of the genes within each group were more similar within groups than among groups. In Group Ⅰ, four *GhNCED* genes (*GhNCED1*, *GhNCED3*, *GhNCED5* and *GhNCED13*) exhibited conserved motifs. Among them, the conserved motif of *GhNCED4* is different from other genes, and it may play a different role in ABA synthesis. In Group Ⅱ, Group Ⅲ and Group Ⅳ, the motifs of the *GhNCED* genes were more conservative, suggesting that these genes may have the same function and may have been highly conserved in the course of evolution.

### Chromosome localization of the *GhNCED* gene family

**Fig 5:**
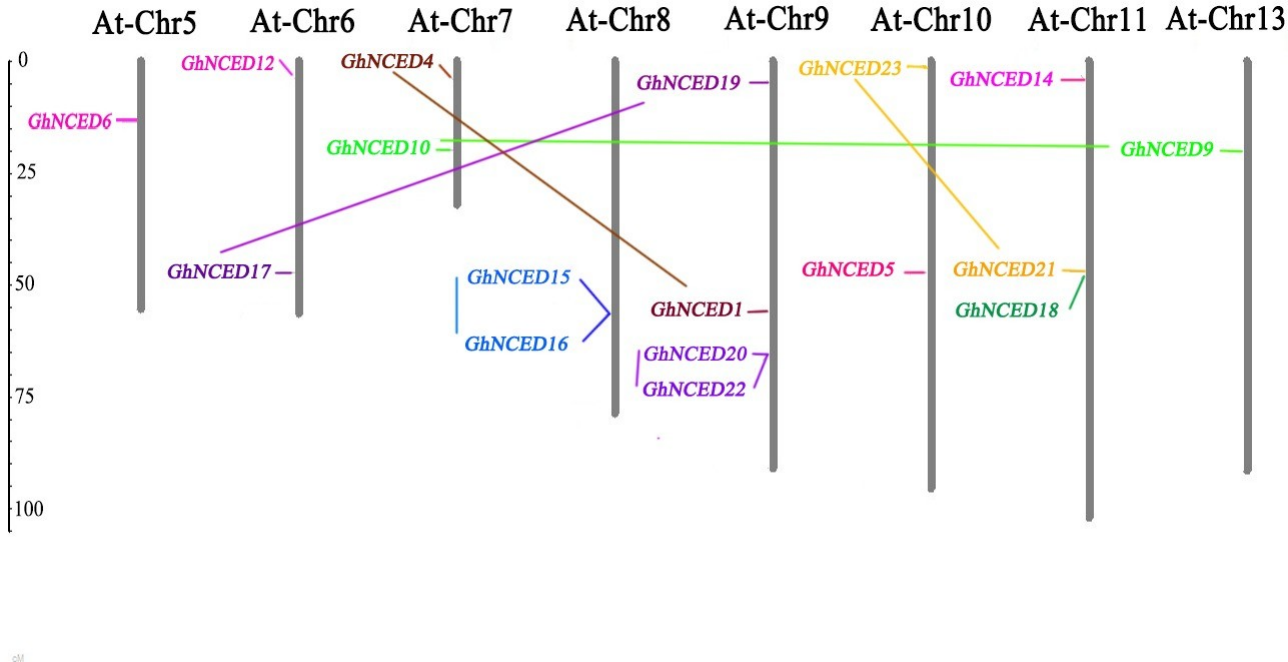
Chromosomal distribution map of the NCED genes in *Gossypium hirsutum* L. The chromosome numbers are indicated at the top of each bar, while the size of a chromosome is indicated by its relative length. The unit of the left scale is Mb, and the short line indicates the approximate position of the *GhNCED* gene on the corresponding chromosome.

Mapinspect software was used to locate 23 *GhNCED* genes on 26 chromosomes of *G*. *hirsutum* (Fig 5). In total, 17 NCED genes were mainly distributed in eight sections of Chr 5, 6, 7, 8, 9, 10, 11 and 13 in *G*. *hirsutum*. Among these chromosomes, Chr 9 contained the largest number (four) of *GhNCED* genes; Chr 11 contained three *GhNCED* genes; Chr 6, 7, 8 and 10 contained two *GhNCED* genes each; and Chr 1 and 13 contained only one *GhNCED* gene each. According to the definition of tandem replication [12, 13], six replication events occurred between *GhNCED* genes.

### Expression analysis of ABA in upland cotton at different stages

**Fig 6:**
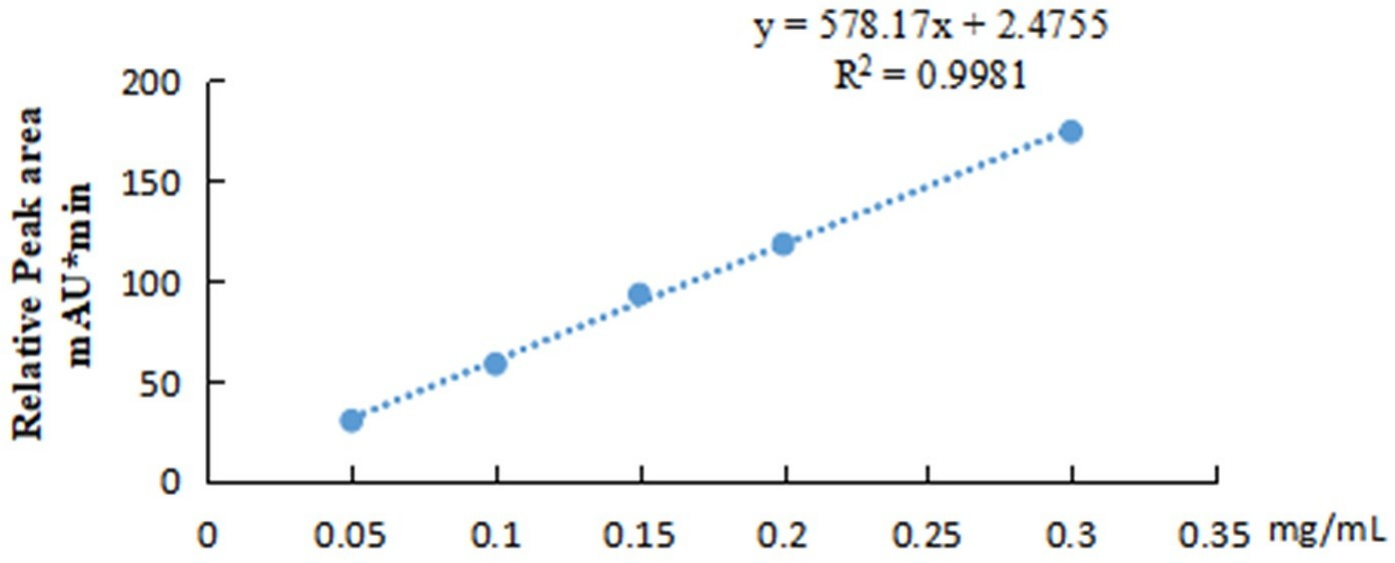
ABA standard curve. ABA solutions with concentrations of 0.05, 0.10, 0.15, 0.20 and 0.30 mg/mL were prepared for HPLC determination. Values represent the mean ± S.E. (n = 3).

**Fig 7:**
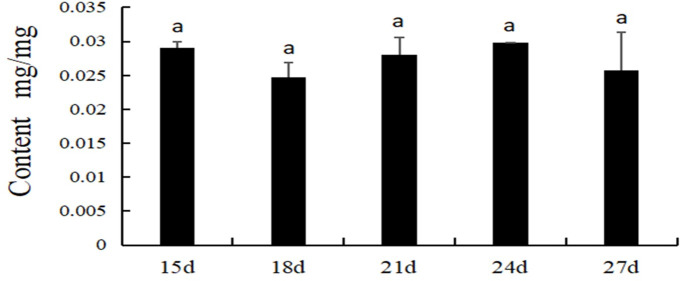
ABA content at different periods in cotton. Values represent the mean ± S.E. (n = 3). Letters indicate significant differences at P < 0.05 according to Duncan’s multiple range tests. Samples were harvested 15, 18, 21, 24 and 27 d after planting.

When the first true leaf grows, the plant is in the seedling stage. Cotton appears in the rapid growth stage. The ABA content was assessed to understand the dynamic changes in ABA in the growth and development stages of cotton. An ABA standard curve was drawn using an ABA standard from SIGMA (Fig 6). ABA solutions at five concentrations (0.05, 0.10, 0.15, 0.20 and 0.30 mg/mL) were prepared for HPLC determination, and an ABA standard curve was obtained. ABA was extracted from cotton leaves every 3 d (Fig 7). The results showed that the ABA content varied among the different stages of upland cotton. The ABA content was high in the early stage, decreased at 18 d and then increased to different extents at 21, 24 and 27 d. However, the ABA content was not significantly different among stages of upland cotton.

### Expression analysis of the *GhNCED* gene family

**Fig 8:**
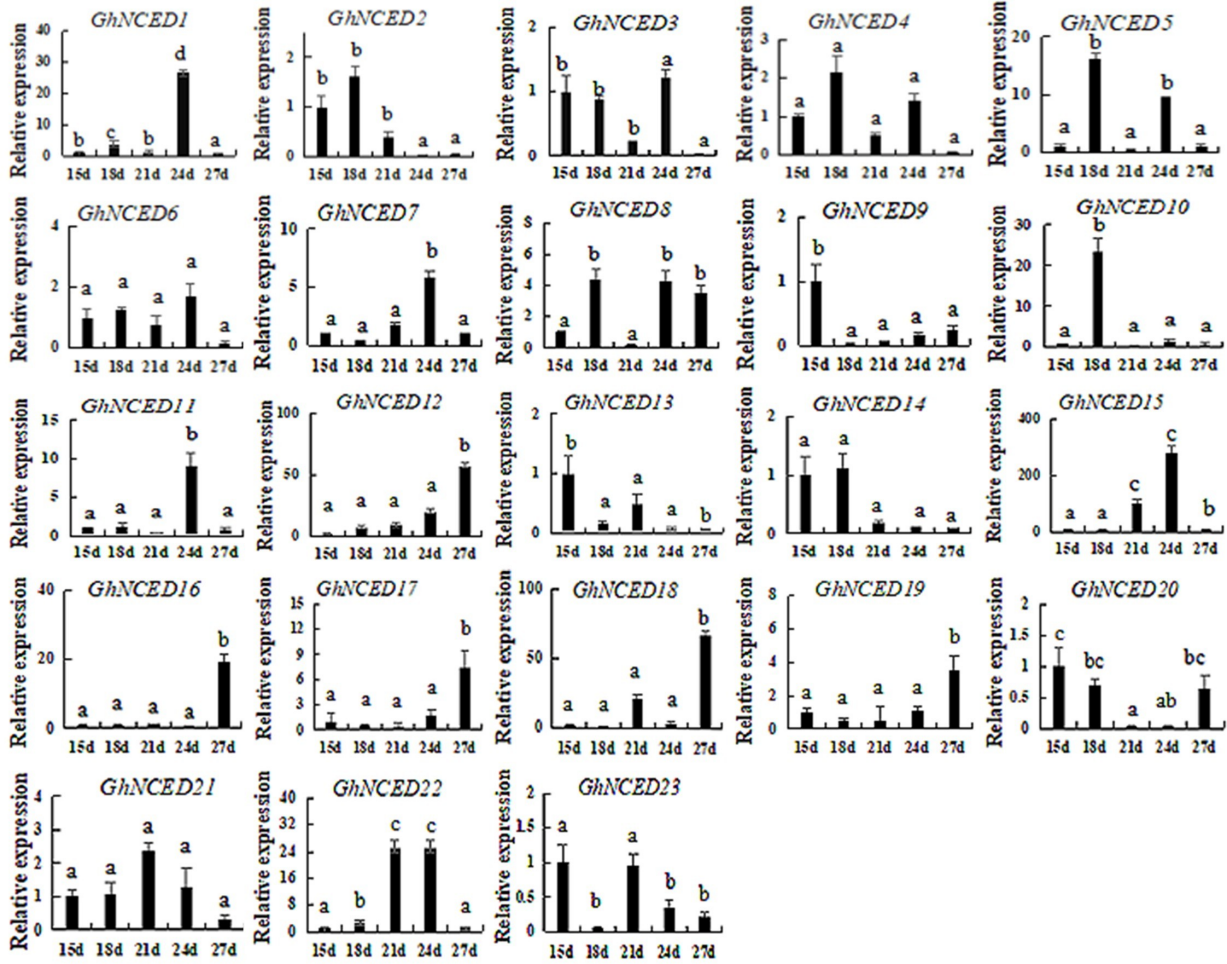
Expression patterns of GhNCED genes at different periods in cotton. Values represent the mean ± S.E. (n = 3). Letters indicate significant differences at P < 0.05 according to Duncan’s multiple range tests. Samples were harvested 15, 18, 21, 24 and 27 d after planting.

To analyze the expression of *GhNCED* genes at five developmental stages, primers for 23 NCED genes were designed (S1 Table), and qRT-PCR was used to study the expression patterns of the five developmental stages at 15, 18, 21, 24 and 27 d after germination (Fig 8). Most of the Group Ⅰ genes exhibited higher expression in the early stage than in the late stage. The change trend of *GhNCED* gene expression was similar to that of ABA content. Group Ⅰ *GhNCED* genes exhibit functions similar to those of *Arabidopsis* NCED genes and directly affect ABA synthesis. Other genes, such as *GhNCED12*, *GhNCED16*, *GhNCED17* and *GhNCED*18, exhibited low expression in the early stage and high expression in the later stage. These results indicate that these genes may function in ABA synthesis inhibition. The expression trends of other genes were more variable, indicating that they play indirect roles in ABA synthesis.

## Discussion

In recent years, a large number of NCEDs have been identified and analyzed in various species using bioinformatics technology. Research progress has been slower for the GhNCED family in cotton than in other species. To date, insufficient information is available about NCEDs in cotton. In the present study, 23 *GhNCED* genes were identified from the cotton genome database.

Gene loss and duplication are the main mechanisms driving species evolution, generating novel gene functions and promoting the formation of gene families. NCED family members have been identified in many plants. Gene duplication and differentiation are the principal pathways for forming new genes and extending gene functions [16]. Members that cluster in similar subgroups commonly exhibit similar functions. Therefore, phylogenetic analysis could facilitate the development of functional genomics. In the present study, we identified 32 NCED proteins with full-length domain sequences from cotton and *Arabidopsis* and classified them into 4 subfamilies on the basis of their sequence structures and phylogenetic relationships.

Phylogenetic trees revealed fourteen GhNCED proteins (60.9%, 14 of 23) in Group I, and five AtNCED proteins (55.6%, 5 of 9) were assigned to this group. These results suggest that the GhNCED proteins in this subgroup may have similar functions in AtNCED proteins. Intron-exon pattern analysis showed that most of the 23 genes contained only one exon and a few contained two or more exons. Numerous reports have shown that the upland cotton genome belongs to an AADD-type tetraploid plant. Although the A and D genomes are highly collinear [17, 18], they have undergone asymmetric evolution, which is well represented in our study. The 23 *GhNCED* genes were distributed on 8 chromosomes of the A genome, which is likely to be the result of the higher gene loss and higher inactivation frequency in the D subgroup than in the A subgroup.

Gene expression profiles often has some connection with gene function. To date, although the patterns of NCED gene expression have been determined in other plants, there have been fewer detailed studies of the expression of cotton NCED genes. In the present study, the expression profiles of *GhNCED* genes in different periods were examined using qRT-PCR, and an HPLC method was established for determination of endogenous ABA content. The ABA content exhibited dynamic changes in a normal environment. The expression pattern of three genes (*GhNCED5*, *GhNCED6* and *GhNCED13*) was similar to that of ABA, which may play a role in regulating ABA synthesis. Our study provides a theoretical foundation for further research on NCED genes, such as investigations of the mechanisms by which NCED genes respond to ABA signaling to regulate the growth and development of upland cotton.

## Conclusions

In this study, a total of 23 *GhNCED* genes were identified from the cotton genome database, and the phylogenetic relationships, exon-intron structure, conserved motifs, chromosomal localization, gene expression profiles and ABA content were comprehensively evaluated under normal growth. The bioinformatics results and analysis of NCED gene expression profiles provide a useful foundation for future studies aimed at understanding the potential role of each GhNCED gene in regulating growth and development of *Gossypium hirsutum* L.

## Supporting information

S1 Table Primer sequences. (DOCX)
