# Supplementary material for: Genome-wide identification and expression analysis of the NCED family in cotton (Gossypium hirsutum L.)
Source: PLoS One. 2021 Feb 25;16(2):e0246021. doi: 10.1371/journal.pone.0246021 (PMC7906304; doi:10.1371/journal.pone.0246021)
Supplement: S1 Table — (DOCX) [file pone.0246021.s001.docx]

**S1 Table. Primer sequences**

| Gene | Forward primer (5′-3′) | Reverse primer (5′-3′) |
| --- | --- | --- |
| UBQ | GAAGGCATTCCACCTGACCAAC | CTTGACCTTCTTCTTCTTGTGCTTG |
| GhNCED1 | AAAACCGCAGACCCACAT | AACAGGGCGACCCAACTC |
| GhNCED2 | ATGGCTTCTTCAACAGGTATTTGGG | CCCAAATACCTGTTGAAGAAGCCAT |
| GhNCED3 | CCGAACAACCATACCAAA | CAATCCCTGAGTGACCAT |
| GhNCED4 | AAACCGCAGACCCACAGG | AACAGGGCGACCCAACTC |
| GhNCED5 | TGGAACGCTTGGGAGGAG | TCGGCAATGGCGAGATAA |
| GhNCED6 | TGGCTTTGGATGTAGTGG | ACTGGTTCGTGAAGTGGG |
| GhNCED7 | TCGCCGTGCCATTATTTC | ACCGTCGTCTTCGTTCTCC |
| GhNCED8 | TGGCTTTGGATGTAGTGG | ACTGGTTCGTGAAGTGGG |
| GhNCED9 | GAGCCAGAAACTGATGAA | TTAGCAAACCCTGACACT |
| GhNCED10 | GGCTTTAGATGTGGTGGA | ACAGGTTCGTGAAGTGGG |
| GhNCED11 | ATCAACAGCAGCATCAGT | AAGAACATGAAATGTAGCG |
| GhNCED12 | ATGCAACCCTCACTTCACTTCTTTA | TAAAGAAGTGAAGTGAGGGTTGCAT |
| GhNCED13 | TGGGAAGAAATATCCGACAC | TTGCTATGCCCGAACACT |
| GhNCED14 | CTAAGTGGAACCTCCTACAA | GTTGATACAAGAAGGGATAGTT |
| GhNCED15 | ATCACCTTATTATGCTCCTC | CAGCCTTGGTTCTACTCTTT |
| GhNCED16 | ATCACCTTATTATGCTCCTC | ACAGCCTTGGTTCTACTCT |
| GhNCED17 | GACGAAATGAAGCCAACC | GACTCGCATAAAGCCAAA |
| GhNCED18 | ACAGACTTGTTCGTGGTA | ACAGACTTGTTCGTGGTA |
| GhNCED19 | AACCCACAACTCCAACCT | TTCCCAAGCATTTACGAT |
| GhNCED20 | GCTTTAGGTGACTGGGATG | AGGTCCTGGTATGGCTGA |
| GhNCED21 | TCACCTCTTTGACGGCTAC | CACCCTTCCAATCACCTT |
| GhNCED22 | AGCCCACTCTTTCATTCT | ACTGTAGCAGCGTTTGTC |
| GhNCED23 | CTCGGCTCATCCCATAGT | TGGTCCGTTCCTACAGTTA |
